# A New Styptic Preparation

**Published:** 1855-08

**Authors:** 


A New Styptic Preparation. (Bull. Gén. de Thérap., 30 Oct., 1854, p. 386.

The following formula is published by M. Hannon :—Benzoic acid, 1 part; sulphate of alumnia and potash, 3 parts; ergotine of bonjean, 3 parts; water, 25 parts. Mix.

The whole is to be boiled for half an hour in a porcelain capsule, constantly stirring, and replacing the evaporated water by hot water. Evaporated with constant agitation to the consistence of an extract, it presents a chocolate-brown color, strongly astringent taste, and an odor of ergotine. According to M. Hannon, it is the most energetic styptic at present known, whether applied externally to the seat of hæmorrhage, or administered internally. For internal use it is sufficient to prescribe the following pills:—Benzoic acid, 1 gramme; pulverized alum, 3 grammes; ergotine of bonjean, 3 grammes. Mix, and make sixteen pills, one to be taken every two hours.—*Med. Chir. Rev.*

